# Supplementary material for: Associations between dietary microbe intake and mortality risk in individuals with sleep disorders: Evidence from NHANES
Source: PLoS One. 2025 Jun 25;20(6):e0326663. doi: 10.1371/journal.pone.0326663 (PMC12193041; doi:10.1371/journal.pone.0326663)
Supplement: S1 File — (DOCX) [file pone.0326663.s004.docx]

library(nhanesR)

library(reshape2)

library(survival)

library(rms)

library(survey)

library(ggplot2)

library(survminer)

library(riskRegression)

setwd('D:///RRR')

x1<-db_demo(years=2005:2014,Year=T,ageyr = 'age',sex=T,eth1 = 'race',edu=T,marital = T,

poverty = 'PIR',wtmec2yr = 'nhs_wt')

x1$nhs_wt<-1/5*x1$nhs_wt

x2<-db_bodyMeasure(data = x1,BMI_kg.m2 = 'BMI')

x3<-diag_alcohol.user(data = x2)

x4<-diag_smoke(data = x3)

x5<-diag_Hypertension(data = x4,yes1 = T)

x6<-diag_DM(data = x5,DM1 = T)

x7<-diag_Hyperlipidemia(data = x6,yes1 = T)

x8<-dex_WorkActivity(data = x7,activity = T)

x9<-db_slq(data = x8,ever_told_by_doctor_have_sleep_disorder = 'disorder')

x10<-db_drtot(data = x9,energy_kcal = T)

x11<-db_mort(data = x10)

x12<-diag_Pregnant(data = x11)

x13<-diag_PHQ9(data = x12,cut = 10)

y<-read.csv(file = 'live.csv')

pad<-nhs_tsv('paq',years = 2005:2006)

y1<-nhs_read(pad,'PAD200:vigorous','PAQ605:vigorous','pad320:moderate','paq620:moderate',Year = F)

mcq<-nhs_tsv('mcq',years = 2005:2014)

y2<-nhs_read(mcq,'mcq220:cancer',Year = F)

m<-Left_Join(x13,y,y1,y2)

d<-read.csv(file = 'live-disorder-mort.csv')

d$agec<-as.factor(d$agec)

d$race<-as.factor(d$race)

d$edu<-as.factor(d$edu)

d$drinking<-as.factor(d$drinking)

d$smoke<-as.factor(d$smoke)

d$activity<-as.factor(d$activity)

d$outcome<-as.factor(d$outcome)

d$c<-as.numeric(d$outcome)

d$sex<-as.factor(d$sex)

d$live<-as.factor(d$live)

d$disorder<-as.factor(d$disorder)

nhs<-svy_design(data = d)

svy_uv.cox(design = nhs,time = 'time',status = 'cvd',x = c('live','disorder'),round = 3)

svy_uv.cox(design = nhs,time = 'time',status = 'cvd',x = c('live','disorder'),round = 3,adjust = c('age','race','edu','marital','PIR'))

svy_uv.cox(design = nhs,time = 'time',status = 'cvd',x = c('live','disorder'),round = 3,adjust = c('age','race','edu','marital','PIR','drinking','smoke','DM','activity','energy_kcal','PHQ9','Hyperlipidemia','cancer'))

svy_tableone(design = nhs,gv = c('all','cvd'),by = 'outcome',round = 3)

svy_uv.cox(design = nhs,time = 'time',status = 'cvd',x = c('outcome','c'),round = 3)

svy_uv.cox(design = nhs,time = 'time',status = 'cvd',x = c('outcome','c'),round = 3,adjust = c('age','race','edu','marital','PIR'))

svy_uv.cox(design = nhs,time = 'time',status = 'all',x = c('outcome','c'),round = 3,adjust = c('age','race','edu','marital','PIR','drinking','smoke','DM','activity','energy_kcal','PHQ9','Hyperlipidemia','cancer','fiber'))

svy_tableone(design = nhs,cv = c('age','PIR','BMI','energy_kcal'),gv = c('sex','race','marital','edu','drinking','smoke','Hypertension','DM','Hyperlipidemia','cancer','activity','PHQ9','disorder','all','cvd'),by = 'live',c_meanPMse = T,total = T,g_N = T,round = 0)

svy_tableone(design = nhs,gv = c('outcome'),by = 'Year',c_meanPMse = T,total = T)

stratum_model(object = nhs,y = 'cvd',x = 'outcome',time = 'time',

stratum ='agec',p = T)
