# Supplementary material for: Associations between dietary microbe intake and mortality risk in individuals with sleep disorders: Evidence from NHANES
Source: PLoS One. 2025 Jun 25;20(6):e0326663. doi: 10.1371/journal.pone.0326663 (PMC12193041; doi:10.1371/journal.pone.0326663)
Supplement: Attachment — Submitted filename: response to reviewers(3.29).docx [file pone.0326663.s006.docx]

# Response to Reviewers

Dear editors and reviewers

We are truly grateful to the reviewers and editors for the time and effort they have dedicated to this manuscript. We have exerted our utmost efforts to revise this manuscript. The questions from the reviewers are presented in italic and bold below, and the revised parts are highlighted in yellow within the text. We sincerely hope that our revisions will meet with your satisfaction. Once again, we would like to express our heartfelt appreciation for your invaluable comments and the opportunity you have provided us to revise the manuscript. Thank you so much!

# Response to Reviewer1

***Q1.Important findings with a large sample on a fundamental topic in everyone's daily life like nutrition. Could you please clarify in the methods why subjects under the age of 20 were included and not 18 as expected?***

Thank you very much for the reviewer’s valuable comments. We adopted this age cutoff following the World Health Organization’s (WHO) Global Burden of Disease Study, where 20 years is frequently used as the threshold for adulthood. Our study analyzes health risks, diseases, and mortality rates across different age groups, and to ensure data comparability and consistency, 20 years is commonly regarded as the onset of adulthood in many analyses. Additionally, some health surveys by the National Center for Health Statistics (NCHS) in the United States also use 20 years as the starting age for the adult group.Secondly, in many regions and states in the United States, individuals over 20 years old are legally allowed to smoke and drink, as stipulated by the Public Health Service Act and the National Minimum Drinking Age Act. Therefore, we excluded individuals under 20 years of age from the analysis.Thank you again for your insightful suggestion!

***Q2.Also in the methods, the assessment of sleep disturbance in the subjects was done by a self-reported questionnaire.This assessment should be done by an official questionnaire like the Pittsburgh Sleep Quality Index***.

We sincerely appreciate the reviewer’s valuable suggestion regarding the use of the Pittsburgh Sleep Quality Index (PSQI) for a more precise and detailed assessment of sleep disorders. However, as the NHANES database is a large and complex public dataset, it does not include specialized questionnaires such as the PSQI. As a result, we had to rely on self-reported physician-diagnosed sleep disorders as a surrogate measure. Fortunately, this approach still aligns with our research objectives, which focus on whether higher dietary microbial intake can mitigate the health risks associated with sleep disturbances. We fully acknowledge the limitations of this method and, if feasible in the future, would gladly follow the reviewer’s recommendation to conduct more detailed investigations using standardized sleep assessment tools. For reference, several prior studies using NHANES data (e.g., PMID: 40069615, PMID: 36464094, PMID: 35277151) have similarly employed sleep disorder questionnaires to achieve their research aims. Once again, we deeply appreciate the reviewer’s insightful comment！

***Q3.In introduction, the relationship between sleep disorders like OSA, bad dietary habits and brain disorders could be improved by the additional references like: Evaluation of wakefulness electroencephalogram in OSA patients. doi: 10.1007/s11325-024-03116-y.***

We sincerely appreciate the reviewer's valuable suggestion regarding the need for additional literature citations in our introduction. In response to your recommendation, we have carefully incorporated the suggested reference into our manuscript. Specifically, we have added it as reference [20] in the following statement: "Epidemiological studies reveal that around 30% to 40% of adults experience varying degrees of sleep disturbances, including insomnia, sleep apnea, or circadian misalignment, often co-occurring with metabolic and psychological conditions [17-20]." This important addition has significantly strengthened the scientific foundation of our paper and enhanced its overall persuasiveness. We are truly grateful for the reviewer's insightful comments, which have undoubtedly improved the quality of our manuscript.

Once again, we extend our deepest appreciation for your constructive feedback and expert guidance.

# Response to Reviewer2

***Q1.The study relies on expert consensus to classify dietary microbial content into broad categories (low, moderate, high) without laboratory-based quantification. This introduces potential inaccuracies in the measurement of actual microbial intake. Such imprecision could lead to misclassification bias, weakening the association between dietary microbial intake and mortality risk.***

We sincerely appreciate the reviewer's valuable comments regarding our methodology. While we have acknowledged this limitation in our discussion, we would like to highlight that the approach we adopted for assessing dietary microbial content has been widely utilized in numerous studies examining its health impacts. This method currently stands as the most convenient, cost-effective, and reliable assessment approach available. Our methodology is based on the established classification system described in:A Classification System for Defining and Estimating Dietary Intake of Live Microbes in US Adults and Children. J Nutr. 2022 Jul 6;152(7):1729-1736. doi: 10.1093/jn/nxac074. PMID: 35583208; PMCID: PMC9258558. Furthermore, we note that this approach has been successfully employed in multiple subsequent studies (PMID: 40098072; PMID: 39756916; PMID: 39615795; PMID: 40026864; PMID: 39738746; PMID: 37827261), further validating its scientific credibility.We are truly grateful for the reviewer's insightful comments. Should more convenient and cost-effective methods for assessing foodborne microbes become available in the future, we would be delighted to incorporate them in our follow-up research. Once again, we deeply appreciate your constructive feedback, which has helped strengthen our manuscript.

***Q2.The study does not control for medications (e.g., antibiotics, probiotics, or sleep aids) or pre-existing gastrointestinal conditions, both of which can significantly alter gut microbiota and influence outcomes.The omission of these factors may confound the relationship between dietary microbes, sleep disorders, and mortality.***

We sincerely appreciate the reviewer's insightful suggestion regarding the potential confounding effects of antibiotics, probiotics, and hypnotics on our results. In response to this valuable comment, we have conducted comprehensive sensitivity analyses as detailed below:"Considering the potential influence of social factors and related medications on dietary live microbe intake and the long-term prognosis of individuals with sleep disorders, this study conducted relevant sensitivity analyses. Key adjustments included social determinants of health (SDOH) [34-35], such as employment status, household income and poverty ratio, food security, education level, access to routine healthcare facilities, health insurance type, home ownership, and marital status; the use of antibiotics, hypnotics (sleeping pills), and probiotic supplements within the past 30 days. The results demonstrated that even after adjusting for these covariates - which may confound the association between dietary microbes and health outcomes in individuals with sleep disorders - those with both sleep disorders and low dietary microbe intake consistently exhibited the highest risks of all-cause mortality and cardiovascular mortality. Thus, the findings remain highly robust, as detailed in Table 6."

However, we acknowledge that our sensitivity analyses did not account for gastrointestinal diseases, primarily due to two limitations of the NHANES database: First, the assessment of digestive disorders relies on subjective self-reports, which may not accurately reflect complex gastrointestinal conditions. Second, data on certain common digestive symptoms (e.g., diarrhea or constipation) were only available for three cycles (6 years), while information on more specific conditions like peptic ulcers was even more limited in coverage. These constraints explain why gastrointestinal factors have rarely been adjusted for in NHANES-based nutritional studies.

We fully recognize this as a study limitation and sincerely appreciate the reviewer's comment, which has helped identify an important area for future research. Should more comprehensive gastrointestinal data become available in subsequent NHANES cycles, we would be pleased to incorporate these analyses in follow-up studies. Once again, we deeply appreciate the reviewer's constructive feedback, which has significantly strengthened our manuscript.

Table 6. Sensitivity analysis of the combined effects of dietary microbes and sleep status on long-term prognosis

| Sleep Disorder | Dietary live microbes | ALL-cause Mortality | | CVD-Cause Mortality | |
| --- | --- | --- | --- | --- | --- |
|  |  | HR (95%CI) | *P* | HR (95%CI) | *P* |
| No | High | ref | ref | ref | ref |
|  | Middle | 0.958(0.819,1.121) | 0.595 | 1.079(0.857,1.358) | 0.516 |
|  | Low | 1.255(1.096,1.438) | 0.001 | 1.405(1.072,1.841) | 0.014 |
| Yes | High | 1.225(0.899,1.669) | 0.199 | 1.663(0.893,3.096) | 0.109 |
|  | Middle | 1.247(0.995,1.562) | 0.055 | 1.044(0.645,1.689) | 0.861 |
|  | Low | 1.673(1.349,2.074) | <0.0001 | 2.161(1.365,3.421) | 0.001 |
| *P* for trend |  | <0.001 | | <0.001 | |

Sensitivity Model: Further adjusted from Model 3 by including hypnotics, probiotic supplements, antibiotics, and SDOH.

***Q3.The use of self-reported data for sleep disorders may introduce recall bias or inaccuracies. Additionally, sleep data is only assessed at baseline, without follow-up to track changes.Subjective sleep data may not accurately capture the severity or nature of sleep disorders, leading to under- or overestimation of their association with mortality.***

We are sincerely grateful for the reviewer's valuable comments regarding the sleep-related data in our study. We would like to address these points as follows：The sleep disorder data in our study were collected through the NHANES questionnaire item "Has a doctor ever told you that you have a sleep disorder?" This represents a professional clinical evaluation of participants' sleep status over an extended period. Moreover, the NHANES working group has implemented standardized quality control procedures to ensure the reliability of these questionnaire data, making them scientifically credible. However, as the reviewer rightly pointed out, this approach does not allow for longitudinal follow-up of individual sleep patterns - a limitation inherent to the cross-sectional design of the NHANES database. We have explicitly acknowledged this limitation in our Discussion section: "First, the classification of dietary live microbes was based on expert consensus, which, though widely cited, may introduce inaccuracies due to the lack of precise microbial quantification in foods. Secondly, the use of self-reports to assess the situation of dietary intake and sleep disorders may not be able to capture the long-term dietary and sleep conditions, resulting in some unnecessary errors."

We would also like to note that this assessment method has been widely adopted in numerous high-quality studies (PMID: 39944955, PMID: 40069615, PMID: 36464094, PMID: 35277151), demonstrating its acceptance in the scientific community. While we fully recognize its limitations, this approach currently represents the most practical, cost-effective, and generally recognized methodology for large-scale epidemiological research of this nature.

We deeply appreciate the reviewer's insightful comments, which have helped us better articulate both the strengths and limitations of our methodology. We hope this response adequately addresses your concerns. Should you have any further questions, we would be happy to provide additional clarification. Once again, thank you for your constructive feedback, which has significantly contributed to improving our manuscript.

***Q4.Dietary data is based on a single 24-hour recall, which may not reflect habitual intake. This short-term dietary snapshot is used to draw conclusions about long-term health outcomes.Dietary habits can fluctuate, and a single-day recall may not accurately represent an individual’s typical intake of live microorganisms***

We sincerely appreciate the reviewer's valuable comments regarding our description of the 24-hour dietary recall methodology. We have revised our manuscript to provide a more detailed explanation as follows:"All dietary microbe intake data were collected through two 24-hour dietary recall interviews. The first dietary recall interview was conducted at the Mobile Examination Center (MEC), while the second interview was performed via telephone 3 to 10 days later. Participants were asked to recall all foods and beverages consumed during the previous 24-hour period (from midnight to midnight). To assist with accurate reporting, participants were provided with a set of measurement guidelines and a food model booklet to help describe food portions and details."

We fully acknowledge the reviewer's valid concern that short-term dietary assessments may not perfectly reflect long-term health impacts. However, this approach currently represents the most practical and cost-effective method available, as conducting longitudinal dietary follow-up for tens of thousands of NHANES participants would be prohibitively expensive in terms of both resources and funding. As noted in our response to Question 1, this methodology has been widely adopted in numerous high-quality studies examining both dietary microbes (PMID: 40098072; PMID: 39756916; PMID: 39615795; PMID: 40026864; PMID: 39738746; PMID: 37827261) and long-term health outcomes using NHANES dietary data (PMID: 39793707, PMID: 39188084, PMID: 39906240, PMID: 39927928). While we recognize the inherent limitations of using short-term dietary assessments to evaluate long-term health effects, the established scientific consensus suggests this remains a valid and widely accepted approach in large-scale epidemiological research.

We are deeply grateful for the time and effort the reviewer has dedicated to evaluating our work. Your insightful comments have significantly strengthened our manuscript. We hope this response adequately addresses your concerns, and we would be pleased to provide any additional clarification if needed. Thank you once again for your valuable feedback and constructive suggestions.

***Q5.While the manuscript suggests mechanisms (e.g., gut-brain axis, neurotransmitter regulation), it does not experimentally validate these pathways. Proposed mechanisms remain speculative without direct evidence, limiting the study’s impact in advancing mechanistic understanding.***

We sincerely appreciate the reviewer's valuable comments regarding our study. We would like to humbly address these important points as follows: Our research represents a clinical cross-sectional study utilizing the NHANES database, and as such, we acknowledge that the conclusions drawn can only establish associations rather than definitive causal relationships. This limitation, along with the absence of animal or cellular experiments that could provide mechanistic insights, has been explicitly discussed in our manuscript's limitations section.

We fully recognize that the current understanding of how dietary microbes and probiotics influence sleep quality and long-term health outcomes remains incomplete. This knowledge gap extends to the fundamental relationships between gut microbiota, sleep, and overall health. A significant challenge in this field stems from the notable differences between animal and human gut microbiomes, meaning that findings from animal studies may not directly translate to human health outcomes. Given these considerations, we believe that clinical studies like ours, while limited in their ability to elucidate underlying mechanisms, represent a practical and cost-effective approach to investigating the potential health impacts of dietary microbes in human populations. Such clinical research can still yield meaningful and clinically relevant observations, despite its inherent methodological constraints.

We are deeply grateful for the time and expertise the reviewer has devoted to evaluating our work. Your thoughtful comments have significantly strengthened our manuscript, and we sincerely hope this response adequately addresses your concerns. Should you require any additional clarification, we would be honored to provide further details. Once again, we express our profound gratitude for your valuable insights and constructive feedback.

***Q6.The study does not account for lifestyle factors like stress, overall diet quality, or social support, which are known to affect sleep and mortality. These unmeasured factors may confound the observed relationships and obscure the role of dietary microorganisms.***

We sincerely appreciate the reviewer's insightful suggestion regarding the potential influence of social support and related factors on the relationship between dietary microbes and health outcomes. In response to this valuable comment, we have incorporated adjustments for Social Determinants of Health (SDoH) in our sensitivity analyses, including: employment status, household income, food security, education level, access to healthcare, health insurance coverage, housing stability, and marital/cohabitation status.The methodology for these adjustments was carefully developed based on two high-quality references: PMID: 37244672 and PMID: 39663346. These studies provided robust frameworks for operationalizing SDoH in epidemiological research.

We are deeply grateful for the reviewer's constructive feedback, which has significantly strengthened our study's rigor and completeness. Your expert comments have been instrumental in improving our manuscript, and we sincerely hope this response adequately addresses your concerns. Once again, we extend our heartfelt appreciation for your time and valuable suggestions, which have greatly enhanced the quality of our work. Should you have any further questions, we would be pleased to provide additional clarification.
