# Supplementary material for: Associations between dietary microbe intake and mortality risk in individuals with sleep disorders: Evidence from NHANES
Source: PLoS One. 2025 Jun 25;20(6):e0326663. doi: 10.1371/journal.pone.0326663 (PMC12193041; doi:10.1371/journal.pone.0326663)
Supplement: Attachment — Submitted filename: Response to reviewer.docx [file pone.0326663.s007.docx]

# Response to Reviewer3

Dear editors and reviewers

We are truly grateful to the reviewers and editors for the time and effort they have dedicated to this manuscript. We have exerted our utmost efforts to revise this manuscript. The questions from the reviewers are presented in italic and bold below, and the revised parts are highlighted in yellow within the text. We sincerely hope that our revisions will meet with your satisfaction. Once again, we would like to express our heartfelt appreciation for your invaluable comments and the opportunity you have provided us to revise the manuscript. Thank you so much!

***Q1. The main flaw I see with the study design is the lack of consideration for the intake of other types of food as potential confounders. For example, there is literature on how an increased intake of dietary fibre (PMID: 38011755), polyphenols (PMID: 37290979), and resistant starch (PMID: 36570138), is associated with decreased mortality. Additionally, a decreased intake of foods such as ultra-processed food (PMID: 35231930) is associated with increased mortality. There could be a confounding relationship between the intake of these types of foods. For example, it could be reasonable to hypothesise that people who were more likely to eat microbe-rich foods would also be more likely to eat a diet high in fibre, or high in polyphenols and resistant starch. Are they also more likely to eat a diet low in processed food? This is particularly important when we consider the complex interaction between live dietary microbes and gut health. Literature suggests that the persistence and ameliorating effects of live microbe through diet are aided with intake of dietary fibre, i.e. “prebiotics” (PMID: 36276830). I feel that looking solely at dietary live microbes without considering other dietary factors is a narrow approach.***

We sincerely appreciate the reviewer for carefully reading our manuscript and raising highly meaningful and valuable questions. We also acknowledge that focusing solely on live dietary microbes without considering other dietary factors represents a narrow approach. To address this, we have added two new variables to the sensitivity analysis in this round of revisions: one is "ultra-processed food intake (PMID: 22990468)" and the other is "dietary fiber intake (PMID: 37805459)". Remarkably, after adjusting for these two variables, the results of our sensitivity analysis remained consistent with the previous findings, and the statistical effects even became more convincing. However, we must apologize that due to the wide variety of polyphenols and resistant starches, the NHANES database does not have detailed records of these two indicators, so we were unable to adjust for them in the sensitivity analysis. For this reason, we mentioned in the discussion section that this is a limitation of our study and hope to gain your understanding. In conclusion, we would like to express our heartfelt gratitude again. The reviewer’s precious suggestions have significantly enhanced the significance of our article’s results, and we are truly grateful for the opportunity to improve our work through this rigorous feedback process.

**Table 6. Sensitivity analysis of the combined effects of dietary microbes and sleep status on long-term prognosis**

| Sleep Disorder | Dietary live microbes | ALL-cause Mortality | | CVD-Cause Mortality | |
| --- | --- | --- | --- | --- | --- |
|  |  | HR (95%CI) | *P* | HR (95%CI) | *P* |
| No | High | ref | ref | ref | ref |
|  | Middle | 0.967(0.824,1.135) | 0.684 | 1.087(0.856,1.381) | 0.495 |
|  | Low | 1.239(1.085,1.414) | 0.002 | 1.405(1.086,1.818) | 0.010 |
| Yes | High | 1.368(0.992,1.887) | 0.056 | 1.676(0.901,3.120) | 0.103 |
|  | Middle | 1.324(1.045,1.676) | 0.020 | 1.156(0.697,1.914) | 0.575 |
|  | Low | 1.666(1.351,2.056) | <0.0001 | 2.230(1.400,3.552) | <0.001 |
| *P* for trend |  | <0.001 | | <0.001 | |

Sensitivity Model: Further adjusted from Model 3 by including hypnotics, probiotic supplements, antibiotics, SDOH, Processed food intake, Dietary fiber intake.

***Q2. Regarding covariates in Table 1:
a. Why were only 3 “race” options included? There are other variables such as non-Hispanic Asian and Hispanic in the NHANES dataset.
b. Please be more specific with the schooling level variables. Does “>high school” mean some college and higher?
c. For smoking, I see that it was defined as “smoking history was defined as having smoked more than 100 cigarettes”, is that in the past year, lifetime?
d. And the drinking history “…was determined by whether the individual had
157 consumed at least one 12-ounce beer, 5-ounce glass of wine, or 1.5-ounce shot of liquor in the past year.” This categorisation does not seem sensitive enough and should be refined into more appropriate categories. For example, someone who consumes only 1-2 glasses of wine in a year should not be in the same category as someone who drinks wine daily.***

We are truly grateful to the reviewers for their meticulous and earnest examination of our manuscript. Regarding question a, although the NHANES dataset does contain data on multiple racial and ethnic groups, after applying NHANES sampling weights to reflect the representativeness of the U.S. population, subgroups other than non-Hispanic White and non-Hispanic Black accounted for a relatively small proportion in the weighted sample; thus, to ensure the readability of the table and focus on major groups with sufficient data, we combined smaller subgroups into "other race" rather than failing to consider other populations. For question b, as you described, ">high school" indeed means "some college and higher," so we have revised ">high school" to "Some college or above" in our baseline and you can see the corresponding changes in Table 1 of the article. Concerning question c, our definition of smoking history is based on whether an individual has smoked more than 100 cigarettes in their lifetime, a method used in many high-quality literatures (PMID：40421042，PMID: 38902748). Regarding question d on the definition of drinkers, we are very thankful to the reviewer for pointing out the ambiguity in our definition, which was caused by a typo—the correct statement should be "Drinking status was defined as at least 12 drinks annually. One drink unit was defined as equivalent to 12 ounces of beer, 5 ounces of wine, or 1.5 ounces of distilled spirits," a classification reported in many literatures (PMID：40413481，PMID：40421042，PMID: 38902748). We have highlighted the corrected content in the manuscript, and once again, we thank the reviewers for their care and attention while apologizing for our oversight.

***Q3. In the results and conclusion of the abstract, the use of “risk” implies a causal link between dietary microbes and mortality which was not investigated in this study. The use of strong, causal language is misleading. I would revise to something implying an association only, as that is what was assessed.***

We sincerely appreciate the reviewers' suggestions, which have enabled us to revise the results and conclusion sections of our abstract, making our manuscript more rigorous. The specific revisions to the abstract are as follows: "Results: This study included 21,233 participants, among whom 2,814 all-cause deaths and 877 cardiovascular deaths were documented. Kaplan-Meier survival analysis revealed a significant association between low dietary microbial intake or sleep disorders and elevated mortality. Cox proportional hazards modeling showed that, among individuals with sleep disorders, those with moderate dietary microbe intake had a lower mortality hazard ratio compared to those with low intake. Conversely, the combination of low dietary microbe intake and sleep disorders was associated with the highest all-cause and cardiovascular mortality. Subgroup and sensitivity analyses demonstrated consistent associations across prespecified strata, with the inverse relationship between dietary live microbe intake and sleep disorder–related mortality remaining robust after adjustment for confounders. Conclusion: Low dietary microbial intake and sleep disorders were independently and jointly associated with higher rates of all-cause and cardiovascular mortality in the population. The observed inverse association between higher dietary microbial intake and mortality outcomes, particularly among individuals with sleep disorders, suggests a potential protective trend." These revisions are also highlighted in yellow throughout the manuscript for clarity.

***Q4. Similarly, in the conclusion of the manuscript (lines 444-447), the sentence “This study demonstrates that a diet low in microorganisms, coupled with sleep disorders, can elevate all-cause and cardiovascular mortality risk, whereas increasing dietary microorganism intake may improve long-term prognosis, particularly reducing mortality risk in those with sleep disorders,” should be rephrased to imply that only an association was found, not a causal relationship.***

We greatly appreciate the reviewer's suggestions, and we have revised the potentially misleading expressions in the sentences as requested. The revised version is as follows:"This study demonstrates that low dietary microorganism intake, when combined with sleep disorders, is associated with higher all-cause and cardiovascular mortality rates, whereas higher dietary microorganism intake correlates with improved long-term prognosis. These findings hold significant public health implications, suggesting that dietary microorganism intake could be emphasized in health and lifestyle recommendations to enhance population health and survival outcomes."Thank you again for your invaluable input—these revisions have indeed strengthened the rigor of our article.

***Q5. The R code associated with analysis should be made available to keep in line with the Data Availability policy and for reproducibility.***

We sincerely appreciate the reviewer's suggestion. This time, we will provide the R script code alongside the manuscript to facilitate the reproducibility of our research findings. Thank you for your guidance, which has enabled us to enhance the transparency and rigor of our work.

***6. The title seems to have a grammatical error, as “might mitigates” is incorrect. I would suggest revising to something more concise.***

We greatly appreciate the reviewer's suggestion. We indeed realized that the grammar in our title might be inaccurate. Here, we have revised the title of the manuscript to: "Associations Between Dietary Microbe Intake and Mortality Risk in Individuals with Sleep Disorders: Evidence from NHANES". Thank you for your careful review, which has helped us improve the precision and professionalism of our manuscript's title.

***Q7. In the introduction (lines 80-82), in the statement, “Notably, recent evidence suggests that timely intake of dietary microorganisms may improve sleep by rebalancing gut microbiota[30],” the reference is for a study that was conducted on rats. This sentence should either be rephrased with that context or referenced with more appropriate literature.***

We deeply appreciate the reviewer's meticulous scrutiny. We have realized that our original literature citations were inappropriate. To address this, we have revised the references and included three clinical studies on probiotics and sleep to support this discussion: PMID: 28948675; PMID: 34444980; PMID: 34589719. We hope this revision meets your expectations and enhances the credibility of our manuscript. Thank you again for your valuable feedback!

***Q8. Again, in the introduction (lines 354-355), the statement “Most studies focus on the impact of probiotics on sleep or disease outcomes” requires references.***

We would like to express our gratitude to the reviewer for the constructive comments. In response, we have supplemented the relevant references in this paragraph: PMID: 36555535 and PMID: 38350465. We trust these additions enhance the depth and credibility of our manuscript. Thank you again for your valuable input, which has been instrumental in improving the quality of our work.
